# 2-But­oxy-*N*-[2-(diethyl­amino)­eth­yl]quinoline-4-carboxamide (dibucaine)

**DOI:** 10.1107/S1600536810045460

**Published:** 2010-11-17

**Authors:** Bernard Van Eerdenbrugh, Phillip E. Fanwick, Lynne S. Taylor

**Affiliations:** a Department of Industrial and Physical Pharmacy, Purdue University, 575 Stadium Mall Drive, West Lafayette, IN 47907, USA; b Laboratory for Pharmacotechnology and Biopharmacy, K.U. Leuven, Gasthuisberg O&N2, Herestraat 49, Box 921, 3000, Leuven, Belgium; c Department of Chemistry, Purdue University, 560 Oval Drive, West Lafayette, IN 47907, USA

## Abstract

The mol­ecular conformation of the title compound, C_20_H_29_N_3_O_2_, is stabilized by an intra­molecular C—H⋯O hydrogen bond. The orientation of the amide group to the ring system is characterized by a C—C—C—O dihedral angle of 137.5 (3)°. In the crystal, inter­molecular N—H⋯O hydrogen bonds between the amide groups form *C*(4) chains running parallel to the *a* axis.

## Related literature

For a monograph on dibucaine, see: Sweetman (2009 ▶). For a comparison of the vasoactivity of dibucaine with other amide and ester local anaesthetics, see: Willatts & Reynolds (1985 ▶). For the initial crystal structure determination of dibucaine hydro­chloride monohydrate, see: Hayward & Donohue (1977 ▶). For the subsequent revision of parameters, bond distances and bond angles, see Donohue & Hayward (1980 ▶). Outlier data were removed using a local program based on the method of Prince & Nicholson (1983 ▶).
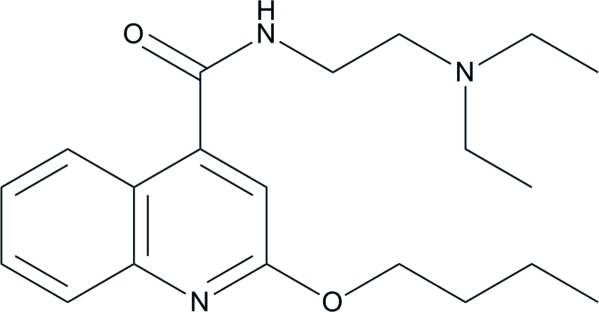


## Experimental

### 

#### Crystal data

C_20_H_29_N_3_O_2_
*M* *_r_* = 343.47
Triclinic, 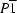

*a* = 4.9323 (1) Å
*b* = 7.2044 (1) Å
*c* = 26.9914 (19) Å
α = 94.080 (7)°
β = 90.611 (6)°
γ = 94.728 (7)°
*V* = 953.30 (7) Å^3^
*Z* = 2
Cu *K*α radiation
μ = 0.62 mm^−1^
*T* = 150 K
0.20 × 0.20 × 0.06 mm

#### Data collection

Rigaku Rapid II diffractometer
Absorption correction: multi-scan (*CrystalClear*; Rigaku, 2001 ▶) *T*_min_ = 0.845, *T*_max_ = 0.966
22671 measured reflections
2786 independent reflections
1829 reflections with *I* > 2σ(*I*)
*R*_int_ = 0.096

#### Refinement

*R*[*F* ^2^ > 2σ(*F* ^2^)] = 0.064
*wR*(*F* ^2^) = 0.168
*S* = 1.05
2786 reflections
234 parameters
H atoms treated by a mixture of independent and constrained refinement
Δρ_max_ = 0.28 e Å^−3^
Δρ_min_ = −0.22 e Å^−3^

### 

Data collection: *CrystalClear* (Rigaku, 2001 ▶); cell refinement: *CrystalClear*; data reduction: *CrystalClear*; program(s) used to solve structure: *SIR2004* (Burla *et al.*, 2005 ▶); program(s) used to refine structure: *SHELXL97* (Sheldrick, 2008 ▶); molecular graphics: *ORTEPII* (Johnson, 1976 ▶) and *PLATON* (Spek, 2009 ▶); software used to prepare material for publication: *SHELXL97* and local programs.

## Supplementary Material

Crystal structure: contains datablocks global, I. DOI: 10.1107/S1600536810045460/rz2498sup1.cif

Structure factors: contains datablocks I. DOI: 10.1107/S1600536810045460/rz2498Isup2.hkl

Additional supplementary materials:  crystallographic information; 3D view; checkCIF report

**Fig. 1.**
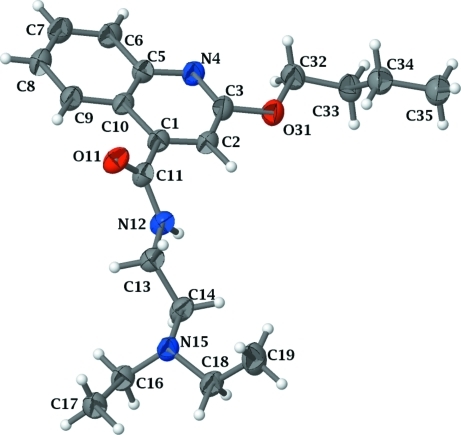
The molecular structure of the title compound, showing 50% probability displacement ellipsoids and the atomic numbering. H atoms are presented as small spheres of arbitrary radius.

Enhanced figure: interactive version of Fig. 1

## Figures and Tables

**Table 1:** Hydrogen-bond geometry (Å, °)

*D*—H⋯*A*	*D*—H	H⋯*A*	*D*⋯*A*	*D*—H⋯*A*
C9—H9⋯O11	0.95	2.43	3.015 (3)	119
N12—H12⋯O11^i^	0.93 (2)	1.93 (2)	2.857 (3)	171 (2)

Symmetry code: (i) 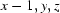.

## supplementary crystallographic information

### Comment

Dibucaine is an amide local anaesthetic that is now generally only used for surface anaesthesia. It is one of the most potent and toxic of the long-acting local anaesthetics and its parenteral use was restricted to spinal anaesthesia (Sweetman, 2009). Although the single-crystal structure of dibucaine hydrochloride monohydrate has been published (Hayward & Donohue, 1977; Donohue & Hayward, 1980), that of the free base has not been reported.

The molecular structure of the title compound is shown in Figure 1. The molecular conformation is stabilized by an intramolecular C—H···O hydrogen bond (Table 1). In the crystal structure, molecules are linked by intermolecular N—H···O hydrogen bonds into chains running parallel to the *a* axis. These hydrogen bonds, formed between the carbonyl oxygen and the amide nitrogen, have a O11···N12 distance of 2.857 (3)Å and a N12—H12···O11 angle of 171 (2)°. In the published structure of dibucaine hydrochloride monohydrate, the hydrogen bonds between the amide groups are disrupted due to hydrogen bonding with chloride and water molecules (Hayward & Donohue, 1977; Donohue & Hayward, 1980).

### Experimental

A non-saturated solution of the title compound was prepared by dissolving the powder to 20 ml of a 1/1 (*v*/*v*) ethanol/water mixture in 20 ml scintillation vials (Research Products International Corp., Mt. Prospect, IL, USA). The open vial was allowed to stand at room temperature to let the liquid slowly evaporate. After one week, the liquid had partly evaporated and crystals of the title compound were obtained. Subsequent to decanting the majority of the remaining liquid and prior to crystal structure determination, the crystals were allowed to dry overnight.

### Refinement

The H atom bound to nitrogen N12 was located in a difference Fourier map and refined freely with isotropic displacement parameters. Other H atoms were placed in calculated positions and treated as riding on their parent atoms with C—H = 0.95 Å (aromatic), 0.99 Å (aliphatic CH_2_), 0.98 Å (aliphatic CH_3_) and with *U*_iso_(H) = 1.2*U*_eq_(C).

### Crystal data

**Table:**

C_20_H_29_N_3_O_2_	*Z* = 2
*M**_r_* = 343.47	*F*(000) = 372
Triclinic, *P*1	*D*_x_ = 1.197 Mg m^−^^3^
Hall symbol: -P 1	Cu *K*α radiation, λ = 1.54184 Å
*a* = 4.9323 (1) Å	Cell parameters from 22671 reflections
*b* = 7.2044 (1) Å	θ = 6–66°
*c* = 26.9914 (19) Å	µ = 0.62 mm^−^^1^
α = 94.080 (7)°	*T* = 150 K
β = 90.611 (6)°	Plate, colourless
γ = 94.728 (7)°	0.20 × 0.20 × 0.06 mm
*V* = 953.30 (7) Å^3^	

### Data collection

**Table:**

Rigaku Rapid II diffractometer	1829 reflections with *I* > 2σ(*I*)
confocal optics	*R*_int_ = 0.096
ω scans	θ_max_ = 66.6°, θ_min_ = 6.5°
Absorption correction: multi-scan (*CrystalClear*; Rigaku, 2001)	*h* = −5→5
*T*_min_ = 0.845, *T*_max_ = 0.966	*k* = −8→8
22671 measured reflections	*l* = −32→32
2786 independent reflections	

### Refinement

**Table:**

Refinement on *F*^2^	H atoms treated by a mixture of independent and constrained refinement
Least-squares matrix: full	*w* = 1/[σ^2^(*F*_o_^2^) + (0.0819*P*)^2^] where *P* = (*F*_o_^2^ + 2*F*_c_^2^)/3
*R*[*F*^2^ > 2σ(*F*^2^)] = 0.064	(Δ/σ)_max_ < 0.001
*wR*(*F*^2^) = 0.168	Δρ_max_ = 0.28 e Å^−^^3^
*S* = 1.05	Δρ_min_ = −0.22 e Å^−^^3^
2786 reflections	Extinction correction: (*SHELXL97*; Sheldrick 2008)
234 parameters	Extinction coefficient: 0.32E-02
0 restraints	

### Special details

**Table:**

Geometry. All e.s.d.'s (except the e.s.d. in the dihedral angle between two l.s. planes) are estimated using the full covariance matrix. The cell e.s.d.'s are taken into account individually in the estimation of e.s.d.'s in distances, angles and torsion angles; correlations between e.s.d.'s in cell parameters are only used when they are defined by crystal symmetry. An approximate (isotropic) treatment of cell e.s.d.'s is used for estimating e.s.d.'s involving l.s. planes.
Refinement. Outlier data were removed using a local program based on the method of Prince and Nicholson (1983).Refinement on *F*^2^ for ALL reflections except for 0 with very negative *F*^2^ or flagged by the user for potential systematic errors. Weighted *R*-factors *wR* and all goodnesses of fit *S* are based on *F*^2^, conventional *R*-factors *R* are based on *F*, with *F* set to zero for negative *F*^2^. The observed criterion of *F*^2^ > σ(*F*^2^) is used only for calculating R_factor_obs *etc*. and is not relevant to the choice of reflections for refinement. *R*-factors based on *F*^2^ are statistically about twice as large as those based on *F*, and *R*- factors based on ALL data will be even larger.

### Fractional atomic coordinates and isotropic or equivalent isotropic displacement parameters (Å^2^)

**Table:**

	*x*	*y*	*z*	*U*_iso_*/*U*_eq_	
O11	0.7912 (3)	0.4509 (2)	0.20477 (6)	0.0463 (5)	
O31	−0.0013 (3)	0.4421 (2)	0.36166 (6)	0.0469 (5)	
N4	0.2537 (4)	0.1888 (3)	0.34861 (7)	0.0416 (6)	
N12	0.3625 (5)	0.5263 (3)	0.19137 (7)	0.0382 (6)	
N15	0.2615 (4)	0.8145 (3)	0.08439 (7)	0.0409 (6)	
C1	0.4443 (5)	0.3476 (3)	0.26107 (8)	0.0362 (7)	
C2	0.2628 (5)	0.4293 (3)	0.29099 (8)	0.0388 (7)	
C3	0.1742 (5)	0.3452 (3)	0.33440 (9)	0.0399 (7)	
C5	0.4357 (5)	0.1009 (3)	0.31830 (8)	0.0392 (7)	
C6	0.5198 (5)	−0.0696 (3)	0.33278 (9)	0.0460 (8)	
C7	0.7017 (5)	−0.1636 (3)	0.30513 (9)	0.0484 (8)	
C8	0.8064 (5)	−0.0917 (3)	0.26159 (9)	0.0473 (8)	
C9	0.7263 (5)	0.0726 (3)	0.24632 (8)	0.0430 (7)	
C10	0.5402 (5)	0.1733 (3)	0.27428 (8)	0.0370 (7)	
C11	0.5499 (6)	0.4428 (3)	0.21690 (9)	0.0384 (7)	
C13	0.4394 (4)	0.6311 (3)	0.14897 (8)	0.0392 (7)	
C14	0.1956 (5)	0.7230 (3)	0.13009 (8)	0.0447 (8)	
C16	0.2090 (5)	0.6808 (3)	0.04102 (8)	0.0456 (8)	
C17	0.3601 (5)	0.7366 (4)	−0.00472 (8)	0.0515 (8)	
C18	0.1034 (5)	0.9769 (3)	0.08026 (8)	0.0489 (8)	
C19	0.2050 (6)	1.1414 (3)	0.11548 (9)	0.0644 (9)	
C32	−0.0885 (5)	0.3722 (4)	0.40823 (8)	0.0475 (8)	
C33	−0.2632 (5)	0.5140 (4)	0.43239 (9)	0.0491 (8)	
C34	−0.1155 (5)	0.7061 (4)	0.44398 (9)	0.0528 (8)	
C35	−0.2952 (5)	0.8435 (4)	0.47007 (9)	0.0603 (9)	
H2	0.1951	0.5431	0.2828	0.047*	
H6	0.4492	−0.1197	0.3621	0.055*	
H7	0.7576	−0.2782	0.3154	0.058*	
H8	0.9337	−0.1576	0.2426	0.057*	
H9	0.7967	0.1193	0.2166	0.052*	
H12	0.180 (5)	0.502 (3)	0.1994 (7)	0.043 (8)*	
H13A	0.5062	0.5461	0.1222	0.047*	
H13B	0.5883	0.7278	0.1588	0.047*	
H14A	0.0410	0.6274	0.1235	0.054*	
H14B	0.1401	0.8165	0.1558	0.054*	
H16A	0.0113	0.6679	0.0333	0.055*	
H16B	0.2619	0.5571	0.0495	0.055*	
H17A	0.3099	0.8593	−0.0133	0.077*	
H17B	0.3120	0.6441	−0.0325	0.077*	
H17C	0.5565	0.7427	0.0019	0.077*	
H18A	−0.0895	0.9409	0.0873	0.059*	
H18B	0.1126	1.0150	0.0458	0.059*	
H19A	0.1885	1.1064	0.1498	0.097*	
H19B	0.0961	1.2467	0.1107	0.097*	
H19C	0.3962	1.1776	0.1087	0.097*	
H32A	0.0708	0.3571	0.4298	0.057*	
H32B	−0.1943	0.2495	0.4024	0.057*	
H33A	−0.3349	0.4670	0.4637	0.059*	
H33B	−0.4205	0.5264	0.4101	0.059*	
H34A	−0.0502	0.7562	0.4126	0.063*	
H34B	0.0456	0.6940	0.4653	0.063*	
H35A	−0.4621	0.8485	0.4504	0.090*	
H35B	−0.1972	0.9678	0.4736	0.090*	
H35C	−0.3419	0.8026	0.5030	0.090*	

### Atomic displacement parameters (Å^2^)

**Table:**

	*U*^11^	*U*^22^	*U*^33^	*U*^12^	*U*^13^	*U*^23^
O11	0.0350 (13)	0.0471 (12)	0.0590 (11)	0.0077 (10)	0.0081 (9)	0.0141 (9)
O31	0.0541 (13)	0.0469 (12)	0.0426 (10)	0.0170 (10)	0.0117 (9)	0.0061 (8)
N4	0.0475 (16)	0.0351 (13)	0.0434 (13)	0.0079 (12)	0.0037 (10)	0.0048 (10)
N12	0.0306 (15)	0.0407 (14)	0.0451 (13)	0.0037 (12)	0.0073 (11)	0.0138 (10)
N15	0.0500 (16)	0.0332 (13)	0.0413 (12)	0.0107 (11)	0.0057 (10)	0.0062 (10)
C1	0.0364 (18)	0.0312 (15)	0.0414 (14)	0.0040 (13)	0.0002 (12)	0.0035 (12)
C2	0.0416 (19)	0.0334 (16)	0.0425 (15)	0.0088 (14)	0.0002 (13)	0.0038 (12)
C3	0.0433 (18)	0.0366 (16)	0.0405 (15)	0.0098 (14)	0.0054 (12)	−0.0001 (12)
C5	0.0443 (19)	0.0334 (16)	0.0402 (15)	0.0069 (14)	−0.0013 (13)	0.0022 (12)
C6	0.058 (2)	0.0334 (16)	0.0485 (16)	0.0103 (15)	0.0027 (14)	0.0082 (13)
C7	0.056 (2)	0.0331 (16)	0.0567 (18)	0.0077 (15)	−0.0021 (14)	0.0067 (13)
C8	0.051 (2)	0.0371 (17)	0.0542 (17)	0.0118 (15)	0.0030 (14)	−0.0003 (13)
C9	0.0460 (19)	0.0375 (16)	0.0471 (16)	0.0110 (14)	0.0042 (13)	0.0054 (13)
C10	0.0361 (18)	0.0312 (15)	0.0439 (15)	0.0059 (13)	−0.0032 (12)	0.0014 (12)
C11	0.0392 (19)	0.0321 (16)	0.0449 (15)	0.0077 (14)	0.0025 (13)	0.0029 (12)
C13	0.0326 (17)	0.0407 (16)	0.0457 (15)	0.0032 (13)	0.0047 (12)	0.0115 (13)
C14	0.0450 (19)	0.0412 (16)	0.0509 (16)	0.0121 (14)	0.0054 (13)	0.0128 (13)
C16	0.051 (2)	0.0357 (16)	0.0503 (17)	0.0066 (15)	0.0008 (14)	0.0038 (13)
C17	0.064 (2)	0.0452 (18)	0.0468 (16)	0.0125 (16)	0.0016 (14)	0.0019 (13)
C18	0.058 (2)	0.0378 (17)	0.0535 (17)	0.0161 (16)	0.0037 (14)	0.0078 (14)
C19	0.095 (3)	0.0378 (18)	0.0614 (19)	0.0118 (18)	0.0086 (17)	−0.0013 (15)
C32	0.054 (2)	0.0491 (18)	0.0405 (15)	0.0085 (16)	0.0092 (13)	0.0054 (13)
C33	0.050 (2)	0.0524 (19)	0.0454 (16)	0.0082 (16)	0.0104 (13)	−0.0003 (14)
C34	0.052 (2)	0.0502 (19)	0.0555 (18)	0.0071 (17)	0.0013 (14)	−0.0036 (15)
C35	0.061 (2)	0.056 (2)	0.0640 (18)	0.0135 (17)	0.0046 (15)	−0.0045 (15)

### Geometric parameters (Å, °)

**Table:**

O11—C11	1.236 (2)	C13—H13B	0.9900
O31—C3	1.348 (2)	C14—H14A	0.9900
O31—C32	1.444 (2)	C14—H14B	0.9900
N4—C3	1.304 (3)	C16—C17	1.511 (3)
N4—C5	1.383 (3)	C16—H16A	0.9900
N12—C11	1.352 (3)	C16—H16B	0.9900
N12—C13	1.452 (3)	C17—H17A	0.9800
N12—H12	0.94 (2)	C17—H17B	0.9800
N15—C14	1.466 (3)	C17—H17C	0.9800
N15—C18	1.469 (2)	C18—C19	1.513 (3)
N15—C16	1.469 (3)	C18—H18A	0.9900
C1—C2	1.354 (3)	C18—H18B	0.9900
C1—C10	1.444 (3)	C19—H19A	0.9800
C1—C11	1.494 (3)	C19—H19B	0.9800
C2—C3	1.414 (3)	C19—H19C	0.9800
C2—H2	0.9500	C32—C33	1.508 (3)
C5—C6	1.408 (3)	C32—H32A	0.9900
C5—C10	1.417 (3)	C32—H32B	0.9900
C6—C7	1.365 (3)	C33—C34	1.520 (3)
C6—H6	0.9500	C33—H33A	0.9900
C7—C8	1.404 (3)	C33—H33B	0.9900
C7—H7	0.9500	C34—C35	1.523 (3)
C8—C9	1.368 (3)	C34—H34A	0.9900
C8—H8	0.9500	C34—H34B	0.9900
C9—C10	1.409 (3)	C35—H35A	0.9800
C9—H9	0.9500	C35—H35B	0.9800
C13—C14	1.520 (3)	C35—H35C	0.9800
C13—H13A	0.9900		
			
C3—O31—C32	117.98 (18)	H14A—C14—H14B	108.10
C3—N4—C5	116.6 (2)	N15—C16—C17	113.6 (2)
C11—N12—C13	120.9 (2)	N15—C16—H16A	108.90
C11—N12—H12	117.6 (13)	C17—C16—H16A	108.90
C13—N12—H12	121.0 (13)	N15—C16—H16B	108.90
C14—N15—C18	110.81 (18)	C17—C16—H16B	108.90
C14—N15—C16	109.95 (18)	H16A—C16—H16B	107.70
C18—N15—C16	110.41 (18)	C16—C17—H17A	109.50
C2—C1—C10	118.5 (2)	C16—C17—H17B	109.50
C2—C1—C11	119.8 (2)	H17A—C17—H17B	109.50
C10—C1—C11	121.6 (2)	C16—C17—H17C	109.50
C1—C2—C3	120.0 (2)	H17A—C17—H17C	109.50
C1—C2—H2	120.00	H17B—C17—H17C	109.50
C3—C2—H2	120.00	N15—C18—C19	112.7 (2)
N4—C3—O31	121.0 (2)	N15—C18—H18A	109.10
N4—C3—C2	124.6 (2)	C19—C18—H18A	109.10
O31—C3—C2	114.4 (2)	N15—C18—H18B	109.10
N4—C5—C6	117.2 (2)	C19—C18—H18B	109.10
N4—C5—C10	123.7 (2)	H18A—C18—H18B	107.80
C6—C5—C10	119.2 (2)	C18—C19—H19A	109.50
C7—C6—C5	120.6 (2)	C18—C19—H19B	109.50
C7—C6—H6	119.70	H19A—C19—H19B	109.50
C5—C6—H6	119.70	C18—C19—H19C	109.50
C6—C7—C8	120.3 (2)	H19A—C19—H19C	109.50
C6—C7—H7	119.90	H19B—C19—H19C	109.50
C8—C7—H7	119.90	O31—C32—C33	106.61 (19)
C9—C8—C7	120.4 (2)	O31—C32—H32A	110.40
C9—C8—H8	119.80	C33—C32—H32A	110.40
C7—C8—H8	119.80	O31—C32—H32B	110.40
C8—C9—C10	120.6 (2)	C33—C32—H32B	110.40
C8—C9—H9	119.70	H32A—C32—H32B	108.60
C10—C9—H9	119.70	C32—C33—C34	114.2 (2)
C9—C10—C5	118.9 (2)	C32—C33—H33A	108.70
C9—C10—C1	124.4 (2)	C34—C33—H33A	108.70
C5—C10—C1	116.6 (2)	C32—C33—H33B	108.70
O11—C11—N12	121.4 (2)	C34—C33—H33B	108.70
O11—C11—C1	123.5 (2)	H33A—C33—H33B	107.60
N12—C11—C1	115.1 (2)	C33—C34—C35	112.7 (2)
N12—C13—C14	109.85 (19)	C33—C34—H34A	109.10
N12—C13—H13A	109.70	C35—C34—H34A	109.10
C14—C13—H13A	109.70	C33—C34—H34B	109.10
N12—C13—H13B	109.70	C35—C34—H34B	109.10
C14—C13—H13B	109.70	H34A—C34—H34B	107.80
H13A—C13—H13B	108.20	C34—C35—H35A	109.50
N15—C14—C13	110.76 (19)	C34—C35—H35B	109.50
N15—C14—H14A	109.50	H35A—C35—H35B	109.50
C13—C14—H14A	109.50	C34—C35—H35C	109.50
N15—C14—H14B	109.50	H35A—C35—H35C	109.50
C13—C14—H14B	109.50	H35B—C35—H35C	109.50
			
C10—C1—C2—C3	0.8 (4)	C2—C1—C10—C9	179.3 (2)
C11—C1—C2—C3	−176.4 (2)	C11—C1—C10—C9	−3.5 (4)
C5—N4—C3—O31	−179.5 (2)	C2—C1—C10—C5	−0.2 (3)
C5—N4—C3—C2	−0.5 (4)	C11—C1—C10—C5	176.9 (2)
C32—O31—C3—N4	2.9 (4)	C13—N12—C11—O11	−0.8 (4)
C32—O31—C3—C2	−176.12 (19)	C13—N12—C11—C1	177.23 (19)
C1—C2—C3—N4	−0.5 (4)	C2—C1—C11—O11	137.5 (3)
C1—C2—C3—O31	178.5 (2)	C10—C1—C11—O11	−39.6 (4)
C3—N4—C5—C6	−179.0 (2)	C2—C1—C11—N12	−40.5 (3)
C3—N4—C5—C10	1.2 (4)	C10—C1—C11—N12	142.4 (2)
N4—C5—C6—C7	−179.2 (2)	C11—N12—C13—C14	−175.3 (2)
C10—C5—C6—C7	0.6 (4)	C18—N15—C14—C13	−149.9 (2)
C5—C6—C7—C8	−0.4 (4)	C16—N15—C14—C13	87.7 (2)
C6—C7—C8—C9	−0.3 (4)	N12—C13—C14—N15	−174.54 (18)
C7—C8—C9—C10	0.8 (4)	C14—N15—C16—C17	−159.16 (19)
C8—C9—C10—C5	−0.5 (4)	C18—N15—C16—C17	78.3 (2)
C8—C9—C10—C1	180.0 (2)	C14—N15—C18—C19	74.0 (3)
N4—C5—C10—C9	179.6 (2)	C16—N15—C18—C19	−164.0 (2)
C6—C5—C10—C9	−0.2 (4)	C3—O31—C32—C33	175.6 (2)
N4—C5—C10—C1	−0.8 (4)	O31—C32—C33—C34	−61.5 (3)
C6—C5—C10—C1	179.4 (2)	C32—C33—C34—C35	−177.9 (2)

### Hydrogen-bond geometry (Å, °)

**Table:**

*D*—H···*A*	*D*—H	H···*A*	*D*···*A*	*D*—H···*A*
C9—H9···O11	0.95	2.43	3.015 (3)	119
N12—H12···O11^i^	0.93 (2)	1.93 (2)	2.857 (3)	170.9 (17)

Symmetry codes: (i) *x*−1, *y*, *z*.
